# Adaptive Structural and Transcriptional Responses Contribute to Cold Tolerance Variation in Xinluzhong 61 and Tahe 2 Cotton Cultivars

**DOI:** 10.3390/ijms27146401

**Published:** 2026-07-18

**Authors:** Hemeng Wang, Yuhao Hu, Fengjiao Wang, Mengmeng Jia, Ziyi Yang, Zhijie Wang, Fuling Wang, Yanqin Wang

**Affiliations:** 1 Key Laboratory of Conservation and Utilization of Biological Resources in the Tarim Basin, Alar 843300, China; hemengwang@yeah.net (H.W.); 15938320634@163.com (Y.H.); 120220045@taru.edu.cn (F.W.);; 2 College of Life Science and Technology, Tarim University, Alar 843300, China; 3 College of Agriculture, Northeast Agricultural University, Harbin 150030, China; 4 Third Division of the Xinjiang Production and Construction Corps Agricultural Research Institute, Tumushuke 843900, China; jiamengmeng_cn@foxmail.com

**Keywords:** cotton, low-temperature stress, RNA-Seq, anatomical investigations

## Abstract

This study elucidated the molecular, morphological and anatomical mechanisms underlying differential cold tolerance between two cotton cultivars, cold-tolerant Xinluzhong 61 (C61) and cold-sensitive Tahe 2 (C2). Seedlings were subjected to 0 °C cold stress for 12 and 24 h, followed by comparative transcriptomic (RNA-Seq) and comprehensive anatomical analyses of cotyledons, true leaves and stems. Transcriptomic profiling identified 8834 and 14,664 differentially expressed genes (DEGs) in C2 and C61 at 12 h, and 14,399 and 16,791 DEGs at 24 h, respectively. KEGG enrichment revealed prominent involvement of phenylpropanoid biosynthesis, photosynthesis, and cutin/suberin/wax biosynthesis at 12 h, shifting to phagosome, cysteine and methionine metabolism at 24 h. Phenotypic and anatomical observations confirmed that C61 developed dense stem glandular trichomes absent in C2, maintained significantly greater leaf thickness, palisade tissue thickness and palisade-to-spongy ratio in true leaves after 24 h stress, and exhibited thicker stem xylem. These findings highlight core adaptive traits and provide valuable genetic targets for improving cold tolerance in cotton.

## 1. Introduction

Cotton, a member of the Malvaceae family, stands as one of the most economically significant crops globally, providing the primary source of natural fiber for the textile industry and contributing substantially to the production of edible oil and animal feed [1,2,3]. Originating from tropical and subtropical regions, cultivated cotton species, particularly upland cotton (*Gossypium hirsutum* L.), are inherently sensitive to a range of abiotic stresses, which pose a significant threat to their growth, development, and productivity [4,5]. Among these environmental challenges, low-temperature or cold stress is a major limiting factor, especially during the critical stages of seed germination and seedling establishment [6]. Exposure to suboptimal temperatures can trigger a cascade of detrimental physiological and biochemical responses, leading to stunted growth, reduced biomass accumulation, impaired reproductive development, and ultimately, significant losses in both fiber yield and quality [7,8].

In major cotton-producing regions such as Xinjiang, China, early-spring cold spells (late spring frosts) frequently disrupt seedling emergence. Chilling temperatures (<15 °C) directly damage the photosynthetic apparatus and induce excessive reactive oxygen species (ROS) accumulation, causing oxidative injury to lipids, proteins and nucleic acids [9]. These cellular disturbances manifest as anatomical alterations, including reduced leaf palisade/spongy mesophyll thickness and impaired vascular structure, further compromising plant function [10,11].

To counteract the deleterious effects of cold stress, plants have evolved a sophisticated and integrated network of defense mechanisms. These adaptive responses span from anatomical modifications to complex biochemical and molecular reprogramming [12]. At the physiological and biochemical level, plants enhance their stress resistance by increasing the activity of antioxidant enzymes, such as Superoxide Dismutase (SOD), Peroxidase (POD), Catalase (CAT). At the molecular level, the perception of a cold signal initiates a signaling cascade that leads to a massive reconfiguration of the transcriptome. This transcriptional reprogramming is central to cold acclimation and involves the differential expression of thousands of genes [13,14]. Particularly, the ICE-CBF-COR pathway is one of the most well-studied pathways in this field [15]. As the core genes of this pathway, CBFs (C-repeat binding factors) are induced by ICEs (inducer of CBF expression) to activate the expression of CORs (cold-responsive genes). Specifically, in Arabidopsis, cold stress induces three CBFs (CBF1, CBF2, CBF3), whereas drought and salt stress trigger CBF4 [16].

Different cotton varieties exhibit considerable differences in their ability to withstand low temperatures, suggesting a genetic basis for this differential tolerance [17]. Identifying the genes and pathways that contribute to the superior performance of cold-tolerant cultivars is a critical step toward developing more resilient cotton varieties through molecular breeding. Comparative transcriptomics, which contrasts the gene expression profiles of tolerant and sensitive genotypes under stress, is a powerful strategy for pinpointing these key genetic determinants. Previous studies in cotton have identified candidate genes involved in cold response, such as those related to lipid metabolism [18], transcription factors [19], and hormone signaling [20].

However, a comprehensive understanding of the integrated mechanisms underlying differential cold tolerance remains elusive. While individual studies have explored either the transcriptomic or anatomical responses in isolation, there is a notable lack of research that systematically combines time-course transcriptomic analysis with anatomical observations across critical seedling organs (e.g., cotyledons, true leaves, and stems) to establish direct links between molecular reprogramming and structural adaptation in cotton. For instance, the link between the transcriptional activation of genes involved in secondary metabolism or structural development and the resulting phenotypic traits, such as altered cell wall composition or the density of protective structures like glandular trichomes, needs to be more firmly established.

To address this gap, In this study, we aimed to dissect the molecular and anatomical basis of differential cold tolerance in cotton. We selected two upland cotton varieties, the cold-tolerant ‘Xinluzhong 61’ (C61) and the cold-sensitive ‘Tahe 2’ (C2), which are widely cultivated in the Xinjiang region of China, an area prone to early-season cold spells. By performing a time-course comparative transcriptomic analysis under severe cold stress (0 °C) and correlating these molecular changes with anatomical adaptations in cotyledons, true leaves, and stems, we sought to identify the key genes, biological processes, and structural modifications that underpin the superior cold tolerance of C61. We hypothesized that the superior cold tolerance of C61 is underpinned by a more rapid and robust activation of specific defense pathways, including antioxidant systems, secondary metabolite biosynthesis, and pathways related to the reinforcement of physical barriers, which would be reflected in both its transcriptomic profile and anatomical features. Furthermore, we investigated the anatomical changes in the cotyledons, true leaves, and stems of both varieties in response to cold stress to correlate the observed transcriptional changes with tangible structural adaptations. By integrating these multi-level analyses, this research aims to provide a comprehensive understanding of the adaptive mechanisms contributing to cold tolerance in cotton, offering valuable insights and potential genetic targets for the future improvement of this vital crop.

## 2. Results

### 2.1. Effects of Low-Temperature Stress on Cotton Seedlings of Xinluzhong 61 and Tahe 2

**Figure 1:**
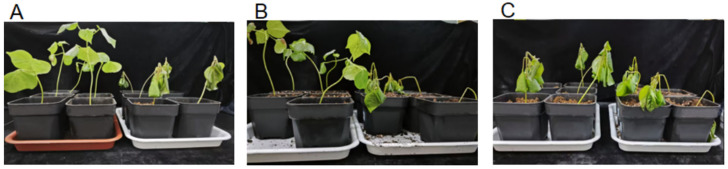
Phenotypes of cotton at different temperatures. Note: (**A**) Xinluzhong 61, left CK, right 0 °C low temperature; (**B**) Tahe 2, left CK, right 0 °C low temperature; (**C**) 0 °C low temperature, left Xinluzhong 61, right Tahe 2.

After 0 °C cold treatment, both cultivars exhibited stress symptoms, but with marked severity differences (Figure 1). C61 seedlings remained upright with only slight leaf wilting and intact stem structure. In contrast, C2 showed severe lodging, extensive leaf wilting, water-soaked lesions and reduced stem toughness. Direct comparison confirmed that C61 maintained significantly better plant morphology and leaf integrity under cold stress, verifying its stronger cold tolerance.

### 2.2. Transcriptome Profiling of Cotton Cultivars C61 and C2 Under Cold Stress

**Figure 2:**
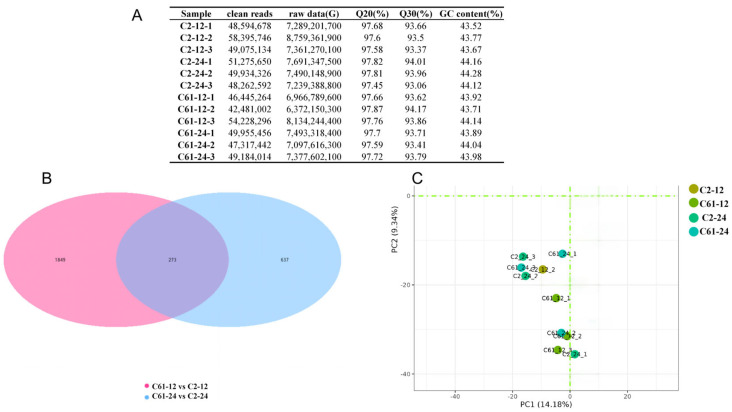
Overview of transcriptome sequencing and sample correlation analysis of Xinluzhong 61 and Tahe 2 cotton cultivars under cold stress. (**A**) Summary of RNA-seq data quality parameters for all samples. (**B**) Venn diagram showing the overlap of differentially expressed genes (DEGs) between cultivar comparisons at 12 h and 24 h of cold stress. (**C**) Principal component analysis (PCA) plot showing the clustering of transcriptomic profiles across all samples.

High-quality RNA-seq data were obtained from two cotton cultivars (C61 and C2) subjected to cold stress for 12 h and 24 h, with clean reads ranged from 42.5 to 58.4 million, Q30 values exceeding 93% and stable GC content (~44%), confirming data reliability for downstream analysis (Figure 2A).

DEG numbers between C61 and C2 increased with stress duration. A total of 273 differentially expressed genes (DEGs) were identified between C61 and C2 at 12 h of cold treatment, whereas 637 DEGs were detected at 24 h (Figure 2B). The marked increase in DEG number at the later time point suggests that prolonged cold stress progressively amplifies transcriptional divergence between the tolerant (C61) and sensitive (C2) cultivars. Ten randomly selected DEGs were quantified by qRT-PCR to validate the RNA-seq results (Table S1).

In addition, principal component analysis (PCA) showed that biological replicates clustered tightly, demonstrating high experimental reproducibility. Samples were primarily separated by treatment duration along PC1 (12 h vs. 24 h), indicating that cold exposure time was the major source of transcriptional variation, with no outliers were observed (Figure 2C).

Taken together, the high-quality sequencing data and clear sample clustering provide a solid foundation for identifying cold-responsive candidate genes and pathways in cotton.

### 2.3. GO Enrichment Analysis of DEGs Induced by Low-Temperature Stress in Two Cotton Varieties

To dissect the molecular mechanisms underlying the differential low-temperature responses of two cotton varieties (Xinluzhong 61, C61, and Tahe 2, C2), we performed Gene Ontology (GO) enrichment analysis on differentially expressed genes (DEGs) under 0 °C treatment for 12 h and 24 h. The DEGs were respectively categorized into C61-12 vs. C2-12 and C61-24 vs. C2-24, with three primary functional categories, as biological process (BP), cellular component (CC), and molecular function (MF).

**Figure 3:**
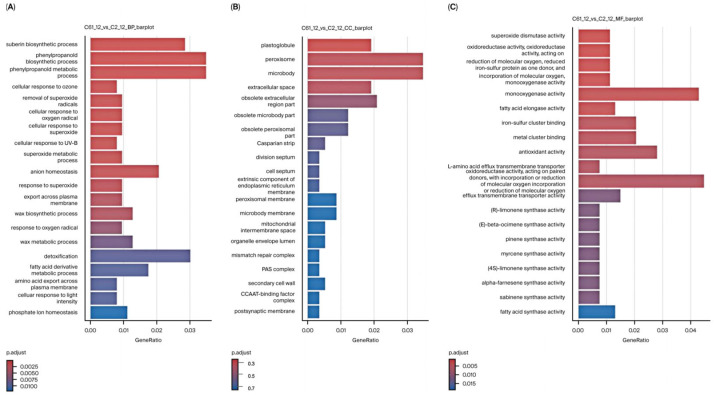
GO analysis of Xinluzhong 61 and Tahe 2 after 12 h of treatment at 0 °C. (**A**) Biological process (BP); (**B**) cellular component (CC); (**C**) molecular function (MF).

At 12 h cold stress, DEGs were significantly enriched in biological processes (BP) related to phenylpropanoid metabolism, ROS response, and cell wall biosynthesis, indicating enhanced stress tolerance and metabolic reprogramming in C61 (Figure 3A). Cellular components (CC) included peroxisome, microbody and plastoglobule, molecular functions (MF) were dominated by oxidoreductase, superoxide dismutase and antioxidant activities, reflecting enhanced ROS detoxification and secondary metabolism in C61 (Figure 3B,C).

**Figure 4:**
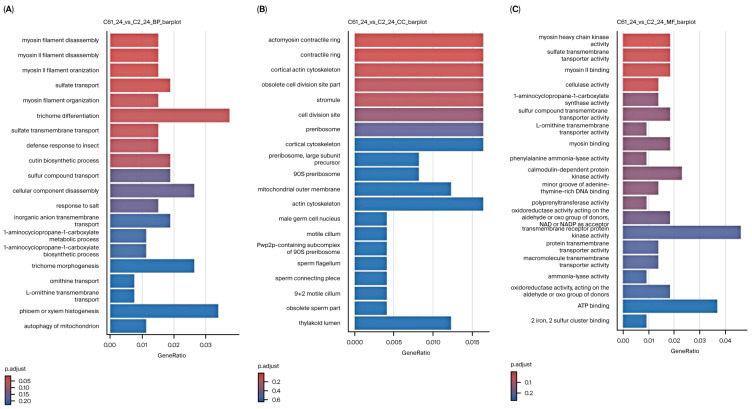
GO analysis of Xinluzhong 61 and Tahe 2 after 24 h of treatment at 0 °C. (**A**) Biological process (BP); (**B**) cellular component (CC); (**C**) molecular function (MF).

At 24 h, BP terms were mainly associated with trichome differentiation, phloem/xylem histogenesis, myosin filament dynamics, sulfur transport, and defense response to insects. CC focused on the actin cytoskeleton and contractile apparatus, as well as ribosome-related components, implying impacts on protein synthesis. MF involved motor activity, transmembrane transport, and catalytic activity, while receptor kinase activity and ATP binding were negatively enriched, suggesting downregulation under prolonged cold stress (Figure 4).

### 2.4. KEGG Enrichment Analysis of DEGs Induced by Low-Temperature Stress in Two Cotton Varieties

To elucidate the key metabolic pathways underlying the differential cold tolerance of these two cotton varieties, KEGG orthology mapping was conducted on the differentially expressed genes (DEGs) under 0 °C treatment (FDR-adjusted *p* < 0.05).

KEGG pathway analysis of DEGs between the two cotton varieties under 12 h of low temperature stress indicated that the most significantly enriched pathways were phenylpropanoid biosynthesis, photosynthesis, cutin, suberine, and wax biosynthesis. Moreover, pathways associated with fatty acid metabolism, terpenoid backbone biosynthesis, and linoleic acid metabolism were also enriched, suggesting that C61 enhances cold tolerance by strengthening cell wall barriers, optimizing energy metabolism, and activating secondary metabolic pathways.

**Figure 5:**
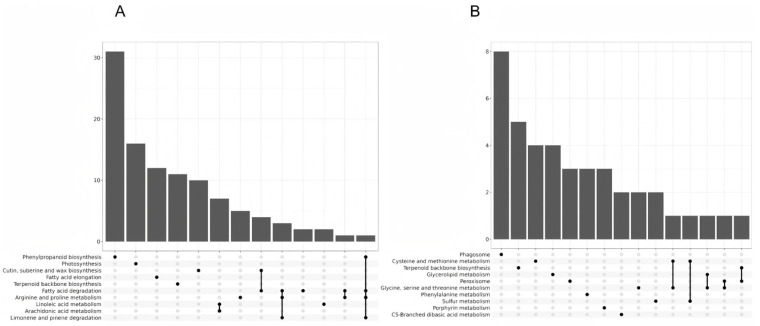
KEGG pathway enrichment analysis of DEGs in Xinluzhong 61 and Tahe 2 after treatment at 0 °C. (**A**) 0 °C treated for 12 h; (**B**) 0 °C treated for 24 h.

In the comparison of the two varieties under 24 h of low temperature stress, DEGs were significantly enriched, with the most prominent enrichment observed in the phagosome pathway, followed by cysteine and methionine metabolism, terpenoid backbone biosynthesis, and glycerolipid metabolism (Figure 5). In contrast to the 12 h treatment, which emphasized phenylpropanoid biosynthesis and photosynthesis, the 24 h treatment shifted towards immune activation and antioxidant metabolism, reflecting a time dependent adaptive strategy for maintaining cold tolerance.

### 2.5. Antioxidant Enzyme Activities in Xinluzhong 61 and Tahe 2 Cotton Cultivars Under Cold Stress

**Figure 6:**
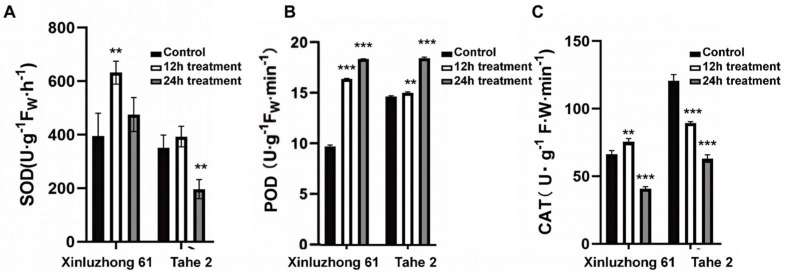
The activities of antioxidant enzymes in Xinluzhong 61 and Tahe 2 under 12 h and 24 h at 0 °C cold treatment. (**A**) SOD activities; (**B**) POD activities; (**C**) CAT activities. Data are presented as mean ± SE. ** *p* < 0.01, *** *p* < 0.001, determined by one-way analysis of variance followed by Tukey’s multiple comparison test.

Due to the enrichment of ROS response in the BP pathway of the transcriptome GO analysis under low temperature, the activities of SOD, POD, and CAT were further measured in Xinluzhong 61 and Tahe 2 (Figure 6). For SOD activity (Figure 6A), Xinluzhong 61 exhibited a rapid and sustained response. For example, activity significantly increased at 12 h (*p* < 0.01) and remained elevated at 24 h, suggesting a robust inducible defense mechanism. In contrast, Tahe 2 showed a delayed but transient response, with a significant decrease at 24 h compared to the control, indicating a compromised SOD-mediated antioxidant capacity under prolonged cold stress. POD activity (Figure 6B) showed a synchronized upregulation pattern in both varieties, with significant increases at 12 h and peak values at 24 h, suggesting that POD serves as a common and critical component of the cold stress response. As shown in Figure 6C, CAT activity revealed distinct temporal dynamics between varieties. Xinluzhong 61 displayed a transient increase at 12 h followed by a sharp decline at 24 h, whereas Tahe 2 maintained significantly higher constitutive CAT activity but showed progressive decreases at both 12 h and 24 h (*p* < 0.001).

These results indicate that Xinluzhong 61 relies on an inducible antioxidant defense system characterized by sustained SOD and transient CAT activation, while Tahe 2 depends more on constitutive CAT activity, revealing divergent antioxidant strategies in response to cold stress.

### 2.6. Effects of Low Temperature Treatment on the Glandular Trichomes of Two Cotton Varieties

**Figure 7:**
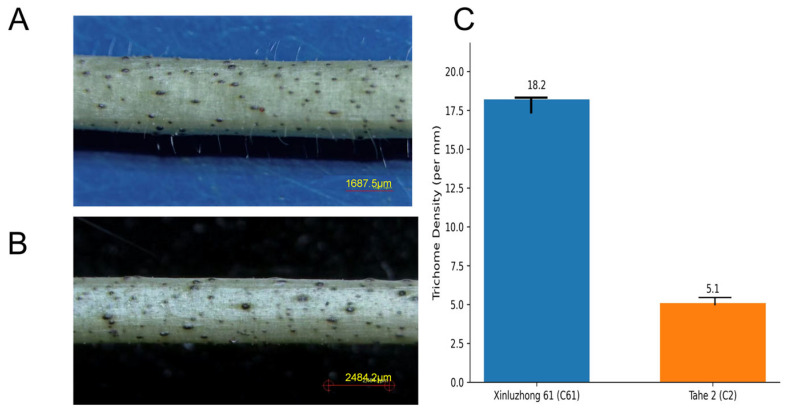
Stem Segment of Xinluzhong 61 and Tahe 2. (**A**) Xinluzhong 61; (**B**) Tahe 2; (**C**) Stem Glandular Trichome Density of Xinluzhong 61 and Tahe 2. Trichome density was counted from at least 10 visual fields per sample with three biological replicates, and statistical significance analysis was performed using ANOVA with Duncan’s post hoc test.

In the biological process category, we noticed that trichome differentiation emerged as the most highly enriched term with the largest gene ratio, indicating that this developmental process was transcriptionally activated in C61 in response to prolonged cold stress. Phenotypic observation of stem tissues revealed striking differences in glandular trichome development between the two cotton varieties under low-temperature stress. As shown in Figure 7, Xinluzhong 61 (C61) exhibited dense and prominent glandular trichomes distributed across the stem surface, whereas Tahe 2 (C2) displayed a relatively smooth stem with significantly fewer trichomes (Figure 7A,B). The quantitative results of trichome density are consistent with our proposed conclusion that trichome differentiation contributes to cotton cold tolerance (Figure 7C). This morphological divergence was strongly supported by our transcriptomic GO enrichment analysis, particularly the results from the 24 h low-temperature treatment.

Collectively, the abundant glandular trichomes in C61 represent a key adaptive phenotype shaped by transcriptional reprogramming. This structural modification, coupled with enhanced secondary metabolism, forms a comprehensive physical and chemical defense system, which likely contributes significantly to the superior low-temperature tolerance of C61 compared to C2.

### 2.7. Cotyledon Anatomical Structure Response Under Cold Stress

**Figure 8:**
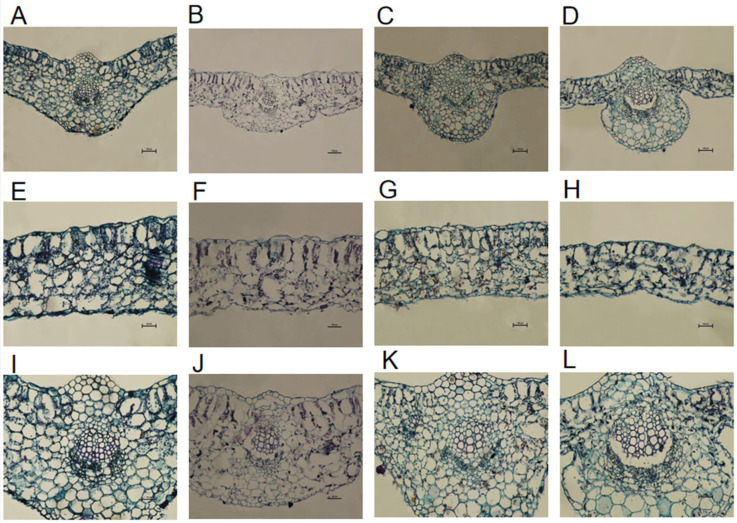
Cold treatment of cotton cotyledon anatomical structure. Note: (**A**) Xinluzhong 61 CK (4×); (**B**) Xinluzhong 61 low temperature at 0 °C for 24 h (4×); (**C**) Tahe 2 CK (4×); (**D**) Tahe 2 low temperature at 0 °C for 24 h (4×); (**E**) Xinluzhong 61 CK (10×); (**F**) Xinluzhong 61 low temperature at 0 °C for 24 h (10×); (**G**) Tahe 2 CK (10×); (**H**) Tahe 2 low temperature at 0 °C for 24 h (10×); (**I**) Xinluzhong 61 CK (10×); (**J**) Xinluzhong 61 low temperature at 0 °C for 24 h (10×); (**K**) Tahe 2 CK (10×); (**L**) Tahe 2 low temperature at 0 °C for 24 h (10×).

Given the distinct and statistically significant variations in leaf morphological traits, we specifically chose the seedlings exposed to 24 h of cold stress for anatomical structure characterization. As shown in Table 1 and Figure 8, low-temperature stress significantly reduced the leaf thickness, palisade tissue thickness, and spongy tissue thickness of cotton cotyledons, and caused rupture in the midrib vascular bundles. Under low-temperature conditions of 0 °C for 12 h, in Xinluzhong 61 leaves, the thickness of palisade tissue, spongy tissue, leaf thickness, palisade-to-spongy ratio, and leaf structural density decreased by 32.14%, 16.15%, 15.55%, 19.15%, and 19.35%, respectively, compared with the control (CK). For Tahe 2 leaves, the thickness of palisade tissue, spongy tissue, and leaf thickness decreased by 22.36%, 35.21%, and 28.78%, respectively, while the palisade-to-spongy ratio and leaf structural density increased by 18.92% and 12%, with differences being statistically significant (*p* < 0.05).

Comparing these two cotton varieties, the cotyledon leaf palisade tissue thickness, spongy tissue thickness, leaf thickness, the palisade–spongy ratio, and leaf structural compactness of Xinluzhong 61 were all higher than those of Tahe 2. This indicates that Xinluzhong 61 has a higher potential photosynthetic capacity and a slower rate of water loss. In the case of low-temperature treatment, the cotyledon leaf palisade tissue thickness, spongy tissue thickness, and leaf thickness of Xinluzhong 61 were also higher than those of Tahe 2, which may be related to the stronger cold tolerance of this variety.

### 2.8. Response of True Leaf Anatomical Structure Under Cold Stress

**Figure 9:**
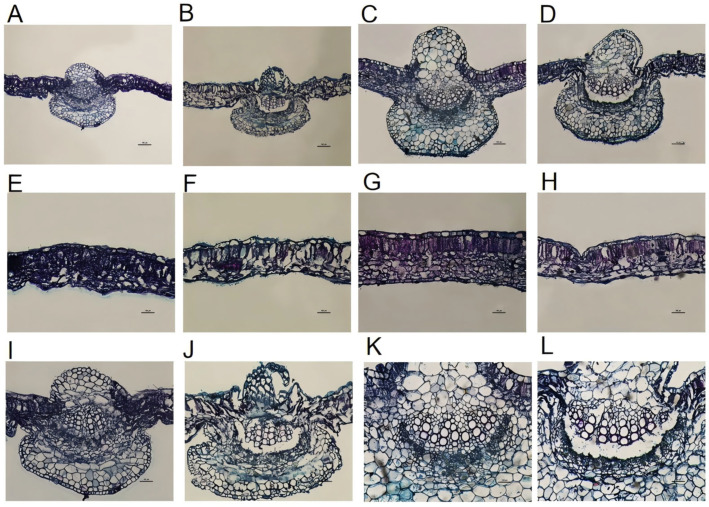
Anatomical structure of true leaves of cotton treated with cold temperature. Note: (**A**) Tahe 2 CK (4×); (**B**) Tahe 2 low temperature at 0 °C for 24 h (4×); (**C**) Xinluzhong 61 CK (4×); (**D**) Xinluzhong 61 low temperature at 0 °C for 24 h (4×); (**E**) Tahe 2 CK (10×); (**F**) Tahe 2 low temperature at 0 °C for 24 h (10×); (**G**) Xinluzhong 61 CK (10×); (**H**) Xinluzhong 61 low temperature at 0 °C for 24 h (10×); (**I**) Tahe 2 CK (10×); (**J**) Tahe 2 low temperature at 0 °C for 24 h (10×); (**K**) Xinluzhong 61 CK (10×); (**L**) Xinluzhong 61 low temperature at 0 °C for 24 h (10×).

Similarly to the cotyledon situation, the effect of low-temperature stress on true leaves is also related to the thickness of the palisade and spongy tissues. As shown in Table 2 and Figure 9, under a low-temperature treatment, the palisade tissue thickness, spongy tissue thickness, and leaf thickness of the Xinluzhong 61 leaf decreased by 20.11%, 42.82%, and 34.47% respectively compared to the control (CK), while the palisade–spongy ratio increased by 40%. For Tahe 2 leaves, the palisade tissue thickness, spongy tissue thickness, and leaf thickness also slightly decreased by 14.73%, 20.58%, and 16.86%, respectively, but the palisade spongy ratio increased by only 7.02%.

The palisade spongy ratio of plant leaves is usually positively correlated with plant stress resistance [21,22]. As seen in Table 2, the palisade tissue thickness, spongy tissue thickness, and leaf thickness of Xinluzhong 61 true leaves were all higher than those of Tahe 2 under normal condition, while there was no significant difference in the palisade spongy ratio (*p* > 0.05) or the leaf structural density between them. After low-temperature treatment, the leaf thickness and palisade–spongy ratio of Xinluzhong 61 leaves were significantly higher than those of Tahe 2, indicating that Xinluzhong 61 has stronger cold resistance than Tahe 2.

Overall, low-temperature stress caused more severe damage to the anatomical structure of Tahe 2 true leaves. This is consistent with the more sensitive low-temperature response observed in phenotypic observations, further revealing the differences in cold tolerance between the two varieties at the anatomical level [23,24].

### 2.9. Response of Stem Anatomical Structure

**Figure 10:**
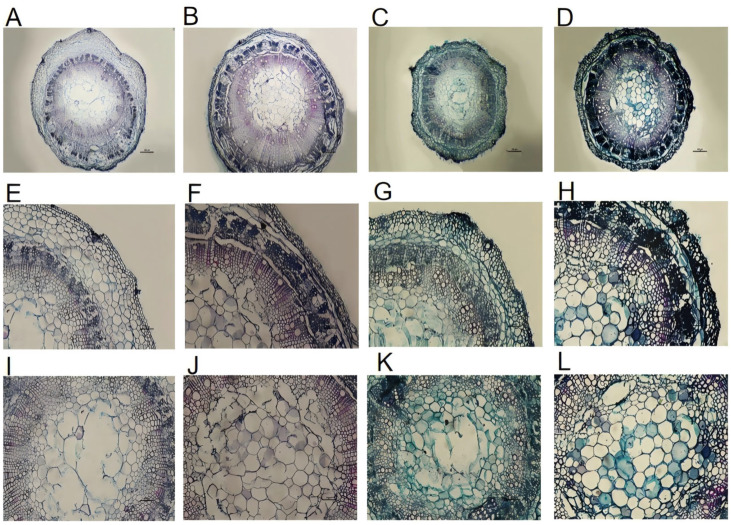
Anatomical structure of cotton stems under low temperature treatment. Note: (**A**) Tahe 2 CK (4×); (**B**) Tahe 2 0 °C low temperature for 24 h (4×); (**C**) Xinluzhong 61 CK (4×); (**D**) Xinluzhong 61 0 °C low temperature for 24 h (4×); (**E**) Tahe 2 CK (10×); (**F**) Tahe 2 0 °C low temperature for 24 h (10×); (**G**) Xinluzhong 61 CK (10×); (**H**) Xinluzhong 61 0 °C low temperature for 24 h (10×); (**I**) Tahe 2 CK (10×); (**J**) Tahe 2 0 °C low temperature for 24 h (10×); (**K**) Xinluzhong 61 CK (10×); (**L**) Xinluzhong 61 0 °C low temperature for 24 h (10×).

Through the analysis of the data in Table 3 and the microstructure in Figure 10, it is evident that after 24 h of 0 °C low-temperature stress treatment, the anatomical structure of cotton stems underwent certain alterations. Specifically, for Xinluzhong 61, the cortex thickness showed a slight decrease of 4.23% compared with the control (CK), while the epidermis, phloem, xylem thicknesses, and pith diameter exhibited increases of 14.97%, 21.14%, 35.98%, and 8.96%, respectively. For Tahe 2, the cortex thickness decreased by 47.17%, the phloem thickness remained relatively stable, and the xylem thickness and pith diameter increased by 19.77% and 12.56%, respectively. Among them, phloem and xylem thicknesses in Xinluzhong 61 exhibited significant statistical differences between the cold-treated and control groups.

It was well-known that xylem is responsible for the upward transport of water and inorganic salts from roots to aerial parts, while phloem transports photosynthates (e.g., sugars) synthesized by leaves to other tissues and organs of the plant. Therefore, plants usually resist adversity by expanding their xylem and phloem. A comparative analysis of the two varieties shows that after low-temperature treatment, the xylem thickness of Xinluzhong 61 increased greater than that of Tahe 2, indicating more tolerance to cold temperature.

## 3. Discussion

Low-temperature stress severely constrains cotton growth, productivity and geographical distribution [25]. Understanding the intricate molecular and physiological mechanisms that underpin cold tolerance is crucial for developing resilient cotton varieties. This study integrated transcriptomic, anatomical and morphological evidence to dissect the multi-level cold-tolerance mechanisms of C61 and C2, demonstrating that C61’s superior cold resilience arises from coordinated temporal metabolic reprogramming and sustained structural adaptation.

The initial response to cold stress is critical for plant survival, as it sets the stage for subsequent acclimation [26]. Our transcriptomic analysis at 12 h post-stress revealed a robust and swift activation of defense-related pathways in the tolerant C61 variety. The significant enrichment of Gene Ontology (GO) terms and Kyoto Encyclopedia of Genes and Genomes (KEGG) pathways associated with phenylpropanoid biosynthesis, reactive oxygen species (ROS) response, and cell wall modifications underscores a proactive defense strategy [27,28]. The phenylpropanoid pathway is a cornerstone of plant defense, producing a vast array of secondary metabolites including lignin, flavonoids, and suberin [29]. The upregulation of this pathway in C61 likely contributes to the reinforcement of cell walls, creating a physical barrier that can limit cellular dehydration and the propagation of ice crystals. Concurrently, the enrichment of pathways for cutin, suberin, and wax biosynthesis further strengthens this physical barrier, reducing non-stomatal water loss and enhancing cellular integrity. This metabolic fortification is complemented by a highly active antioxidant system. The enrichment of GO terms such as oxidoreductase, monooxygenase, and superoxide dismutase activity in C61 points to an enhanced capacity to scavenge ROS, which are inevitably produced under cold stress and can cause significant oxidative damage [30,31,32]. Furthermore, the observed enrichment in fatty acid and linoleic acid metabolism pathways is consistent with previous findings that lipid remodeling is a key component of the cold response in cotton, affecting membrane fluidity and generating signaling molecules like oxylipins [33]. This rapid and multi-pronged metabolic response in C61 likely mitigates the initial shock of cold stress, preserving cellular homeostasis and providing a foundation for long-term survival.

As the duration of cold stress extended to 24 h, the most striking finding from our GO analysis was the dramatic enrichment of the trichome differentiation biological process, which was accompanied by a visible increase in the density of glandular trichomes on the stems of C61. Plant trichomes serve multiple functions in stress tolerance; they can create a microenvironment that insulates the plant surface, reduce water loss by trapping a layer of still air, and, in the case of glandular trichomes, secrete secondary metabolites that can act as chemical protectants [34,35,36]. The development of a dense trichome layer in C61 represents a significant morphological adaptation, forming a physical and chemical barrier against the cold. This finding highlights a less-explored mechanism of cold tolerance in cotton and suggests that developmental plasticity plays a crucial role in long-term acclimation.

Anatomical integrity closely aligns with molecular regulation. Under cold stress, C61 retained significantly greater leaf thickness, as well as thicker palisade and spongy tissues, compared to the sensitive C2 variety. The palisade tissue is the primary site of photosynthesis, and its structural integrity is vital for maintaining energy production under stress [37]. The ability of C61 to preserve and even reinforce its vascular structures underpins its physiological resilience [38]. In contrast, the more severe degradation of leaf and stem tissues in C2 reflects its inability to mount an effective and sustained defense, leading to cellular collapse and functional impairment. This integration of molecular data with anatomical evidence provides a holistic view of cold tolerance [21]. In addition, we also evaluated the expression levels of key genes in the ICE-CBF-COR pathway, including GhICE1, GhCBF1, GhCBF2, GhCBF3, and GhCOR, using RT-qPCR (Supplementary Table S2). As shown in the heatmap, all these genes exhibited remarkably elevated expression under cold stress conditions. Notably, GhCBF1, GhCBF2, GhCBF3, and GhCOR showed strong up-regulation at both 12 h and 24 h of cold treatment, while GhICE1 also displayed increased expression, particularly at 24 h. These results confirm that the ICE-CBF-COR pathway is activated in cotton under low-temperature stress, consistent with its well-established role in cold acclimation across plant species.

The structural plasticity of leaf mesophyll represents a fundamental adaptive mechanism, yet cotyledons and true leaves exhibit distinct remodeling patterns reflecting their divergent developmental origins. True leaves display sophisticated, layer-specific responses. For example, Under salt stress, *Lycium barbarum* shows 49.70% increase at moderate salinity followed by degradation at high concentrations, while spongy tissue initially expands (52.56%) then collapses, optimizing the palisade-to-spongy ratio (PT/ST) at 0.91 [22]. Drought induces 9 to 15% palisade cell diameter reduction with increased bundle sheath extensions in Juglans, coupled with spongy tissue shrinkage and enlarged air spaces to facilitate CO_2_ diffusion [39,40]. Cold-tolerant *Camellia weiningensis* increases palisade thickness and tissue tightness at 4 °C, whereas freezing (−4 °C) disrupts the second palisade layer, causing intermixing with spongy tissue [23].

Cotyledons exhibit limited structural complexity but enhanced metabolic plasticity. *Eruca sativa* cotyledons under salt stress show reduced palisade area but spongy expansion via TOR signaling modulation, with thickness recoverable through metabolic intervention rather than architectural remodeling [24]. This reflects cotyledons’ transient role: they prioritize rapid growth over durable structure, accepting degradation when stress exceeds thresholds. The divergence illustrates adaptive decoupling—true leaves evolved multi-layered complexity for sustained performance, while cotyledons optimize resource mobilization [41]. Domesticated cotton demonstrates how selection shifts the cell size-number trade-off, yielding larger but fewer palisade and spongy cells with thinner walls for enhanced resource acquisition [42].

### Significance, Limitations, and Future Directions

This study significantly advances our understanding of cold tolerance mechanisms in cotton by demonstrating a coordinated, multi-level response in the tolerant variety C61. We have identified not only key metabolic pathways but also a critical role for developmental plasticity, particularly trichome formation, as a key adaptive trait. The differentially expressed genes and pathways uncovered here, such as those controlling phenylpropanoid biosynthesis and trichome differentiation, represent valuable targets for molecular breeding programs aimed at enhancing cold tolerance in elite cotton cultivars [43,44].

However, this study has certain limitations. The findings are based on a comparison of two specific cultivars at discrete time points and a single stress temperature. Future research should expand this analysis to a broader range of cotton germplasm and environmental conditions to validate these findings and uncover additional tolerance mechanisms. A notable limitation is the absence of physiological measurements, which would have provided direct evidence of the physiological status and adaptive responses of cotton plants under cold stress—such as changes in chlorophyll content, photosynthetic rate, electrolyte leakage, or proline accumulation. These physiological parameters are closely linked to the molecular changes observed in our transcriptomic analysis, and their omission means we cannot fully connect transcriptional alterations to actual physiological performance and stress tolerance levels in the studied cultivars. A further limitation is that control samples were collected only at 24 h rather than at both 12 h and 24 h. This single time-point control design represents a potential confound, as temporal changes unrelated to low-temperature stress could not be fully excluded in the comparisons between 12 h/24 h cold-stressed samples and the control. While our transcriptomic analysis provides a powerful snapshot of gene expression, it is correlational. Functional validation of key candidate genes using techniques such as virus-induced gene silencing (VIGS) or CRISPR/Cas9 is necessary to establish their causal roles in cold tolerance [45,46]. Furthermore, integrating proteomics and metabolomics would provide a more complete picture of the molecular landscape, revealing post-transcriptional regulation and the specific biochemical compounds that contribute to protection [47,48]. Investigating the specific chemical composition of the glandular trichome exudates in C61 could also unveil novel protective compounds.

In conclusion, our integrated analysis reveals that the superior cold tolerance of the cotton variety Xinluzhong 61 is orchestrated by a sophisticated and dynamic defense system. This system combines a rapid metabolic response, characterized by the activation of antioxidant and cell wall fortification pathways, with a subsequent phase of morphological adaptation, most notably the development of a dense protective layer of glandular trichomes. These molecular and developmental changes are reflected in the preservation of anatomical integrity, which underpins the plant’s physiological resilience. These findings provide valuable insights into the complex nature of plant stress adaptation and offer promising avenues for the genetic improvement of cold tolerance in cotton.

## 4. Materials and Methods

### 4.1. Plant Materials and Growth Conditions

Two upland cotton (*Gossypium hirsutum* L.) cultivars, Tahe 2 (cold-sensitive) and Xinluzhong 61 (cold-tolerant), were used in this study. Seeds were surface-sterilized with 1% sodium hypochlorite for 10 min, rinsed thoroughly with sterile distilled water, and germinated on moist filter paper in the dark at 28 °C for 48 h. Uniformly germinated seeds were then transferred to plastic pots (10 cm diameter) filled with a sterile mixture of vermiculite and nutrient soil (1:1, *v*/*v*). The seedlings were grown in a controlled environment growth chamber under a 16 h light (28 °C)/8 h dark (22 °C) cycle, with a light intensity of 300 µmol m^−2^ s^−1^ and 60% relative humidity.

### 4.2. Cold Stress Treatment and Sample Collection

When the seedlings reached the three-leaf stage (approximately 21 days after sowing), they were divided into two groups. The control group was maintained under the normal growth conditions described above (28 °C), and control samples were collected only at 24 h, corresponding to the final time point of cold stress treatment. The cold stress group was transferred to another growth chamber set to a constant temperature of 0 °C for 12 and 24 h, with the same light and humidity conditions. For each cultivar and treatment, three biological replicates were established, with each replicate consisting of a pool of five individual seedlings. After the 12 and 24 h treatment period, 7-day-old seedlings were collected from both control and cold-stressed seedlings, and the collected tissue was immediately frozen in liquid nitrogen and stored at −80 °C for RNA extraction.

For trichome density analysis, stem segments was collected from 7-day-old seedlings. Samples were photographed under a stereomicroscope with scale bars. Trichome numbers were counted using ImageJ 1.54r and normalized to stem length (per mm).

### 4.3. RNA Extraction, Library Construction, and Sequencing

Total RNA was extracted from the frozen leaf samples using the TRIzol reagent (Invitrogen, Waltham, MA, USA) according to the manufacturer’s protocol. RNA quality and quantity were assessed using a NanoDrop 2000 spectrophotometer (Thermo Fisher Scientific, Waltham, MA, USA) and an Agilent 2100 Bioanalyzer (Agilent Technologies, Santa Clara, CA, USA). Only RNA samples with an RNA Integrity Number (RIN) > 8.0 were used for library construction. For each sample, 1 µg of total RNA was used to construct a sequencing library using the NEBNext^®^ Ultra™ RNA Library Prep Kit for Illumina^®^ (NEB, Ipswich, MA, USA) following the manufacturer’s instructions. Briefly, mRNA was enriched using oligo(dT)-attached magnetic beads. The enriched mRNA was then fragmented and used as a template for first-strand cDNA synthesis using random hexamer primers. Second-strand cDNA was synthesized using DNA Polymerase I and RNase H. The resulting double-stranded cDNA was end-repaired, A-tailed, and ligated to sequencing adapters. The ligated products were purified and amplified by PCR to create the final cDNA libraries. The quality of the libraries was assessed on the Agilent Bioanalyzer 2100 system. The qualified libraries were sequenced on an Illumina NovaSeq 6000 platform, generating 150 bp paired-end reads.

### 4.4. Functional Annotation and Enrichment Analysis

Raw sequencing reads were first processed to remove adapter sequences, low-quality reads, and reads containing poly-N using Trimmomatic. The quality of the clean reads was verified using FastQC. The clean reads were then aligned to the *G. hirsutum*. TM-1 reference genome (v2.1) using HISAT2 with default parameters. The number of reads mapped to each gene was counted using featureCounts.

Raw read counts were further normalized using the TPM (Transcripts Per Kilobase of exon model per Million mapped reads) method to eliminate the effects of gene length and sequencing depth on expression quantification. Genes with TPM < 1 in more than 50% of the samples were defined as low-expression genes and filtered out before differential expression analysis. Differential expression analysis was performed using the DESeq2 R package (1.42.1), with the screening criteria of |log2(fold change)| ≥ 1 and adjusted *p*-value < 0.05.

To understand the biological functions of the identified DEGs, Gene Ontology (GO) and Kyoto Encyclopedia of Genes and Genomes (KEGG) pathway enrichment analyses were performed using the clusterProfiler R package (v4.12.0). The background gene set was the complete genome sequence of *G. hirsutum* L., and GO terms and KEGG pathways with a padj < 0.05 were considered significantly enriched. Notably, these enrichment analyses were based on DEGs derived from the direct C61 vs. C2 contrast under cold stress (0 °C, 12 h/24 h)—that is, genes exhibiting significant expression differences between the two cultivars after the same cold stress treatment (C61-12 vs. C2-12 and C61-24 vs. C2-24). This approach specifically identifies biological processes and pathways that differentiate the cold-tolerant (C61) and cold-sensitive (C2) varieties under severe cold conditions.

### 4.5. Quantitative Real-Time PCR (qRT-PCR) Validation

To validate the RNA-seq results, the expression levels of ten selected DEGs were quantified by qRT-PCR. First-strand cDNA was synthesized from 1 µg of the same total RNA samples used for sequencing, using the PrimeScript™ RT Reagent Kit with gDNA Eraser (Takara, Kusatsu City, Japan). Gene-specific primers were designed using Primer3Plus. The cotton GhUBQ7 gene was used as the internal reference for normalization. The qRT-PCR reactions were performed on a CFX96 Real-Time PCR Detection System (Bio-Rad, Hercules, CA, USA) using TB Green^®^ Premix Ex Taq™ II (Tli RNaseH Plus) (Takara, Kusatsu City, Japan). The reaction volume was 20 µL, containing 10 µL of TB Green Premix, 0.8 µL of each primer (10 µM), 2 µL of diluted cDNA, and 6.4 µL of nuclease-free water. The thermal cycling conditions were: 95 °C for 30 s, followed by 40 cycles of 95 °C for 5 s and 60 °C for 30 s. The relative expression levels were calculated using the 2^−ΔΔCt^ method. Three technical replicates were performed for each of the three biological replicates.

### 4.6. Paraffin Section Preparation

Cotyledons, the second true leaves and stem segments (1 cm below the cotyledons) were fixed in FAA solution (formalin:acetic acid:70% ethanol, 5:5:90, *v*/*v*/*v*) for at least 24 h at 4 °C. The fixed samples were then dehydrated through a graded ethanol series (70%, 85%, 95%, 100%), cleared with xylene, and embedded in paraffin. Transverse sections of 8 µm thickness were prepared using a rotary microtome (Leica RM2235, Nussloch, Germany). The sections were mounted on glass slides, deparaffinized with xylene, rehydrated through a reverse ethanol series, and stained with Safranin-Fast Green. The stained sections were observed and photographed using a light microscope (Olympus BX53, Tokyo, Japan) equipped with a digital camera. For quantitative analysis, ImageJ software was used to measure various anatomical parameters from at least 10 different sections per biological replicate. The measured parameters included leaf thickness, palisade tissue thickness, spongy tissue thickness, stem diameter, cortex thickness, xylem thickness, and pith diameter.

### 4.7. Statistical Analysis

All statistical analyses for anatomical and qRT-PCR data were performed using SPSS software (version 22.0). Data are presented as the mean ± standard deviation (SD). Significant differences between control and cold-stressed groups were determined using a two-way ANOVA followed by Tukey’s HSD with *p* < 0.05 considered statistically significant.

## 5. Conclusions

This study reveals that cold tolerance in cotton is governed by integrated molecular, morphological and anatomical adaptations. The cold-tolerant cultivar Xinluzhong 61 exhibits time-dependent transcriptional reprogramming: early activation of phenylpropanoid biosynthesis, photosynthesis and cutin/suberin pathways for rapid defense, followed by induction of phagosome and amino-acid metabolism for long-term acclimation. C61 displays more extensive DEG regulation (14,664 vs. 8834 at 12 h; 16,791 vs. 14,399 at 24 h), dense stem glandular trichomes, superior true leaf anatomical structure and thicker stem xylem compared with Tahe 2. These findings clarify the synergistic mechanisms of cotton cold tolerance and provide pivotal genetic targets for molecular breeding of cold-resilient cotton cultivars.

## Figures and Tables

**Table 1:** Effect of Low-Temperature Treatment on the Anatomical Structure of Cotyledons.

Groups	Palisade Tissue Thickness (μm)	Spongy Tissue Thickness (μm)	Leaf Thickness (μm)	Leaf Midrib Thickness (μm)	Palisade-to-Spongy Ratio	Leaf Structural Density
Xinluzhong 61-CK	71.29 ± 7.10 a	153.04 ± 6.5 a	231.63 ± 3.38 a	156.44 ± 17.56 b	0.47 ± 0.03 a	0.31 ± 0.03 a
Xinluzhong 61-Cold	48.38 ± 4.56 b	128.32 ± 6.05 b	195.61 ± 12.85 b	173.88 ± 6.53 b	0.38 ± 0.05 b	0.25 ± 0.02 b
Tahe 2-CK	55.20 ± 5.97 b	149.48 ± 5.44 a	218.94 ± 7.38 a	159.02 ± 2.18 b	0.370 ± 0.03 b	0.25 ± 0.02 b
Tahe 2-Cold	42.86 ± 3.11 c	96.85 ± 11.84 c	155.94 ± 21.48 c	201.78 ± 21.49 a	0.44 ± 0.02 a	0.28 ± 0.03 a

Note: Data are mean ± SD. Different lowercase letters (a, b, c) within the same column indicate significant differences at *p* < 0.05. a = lowest mean, b = highest mean.

**Table 2:** The Effect of Low-Temperature Treatment on the Anatomy of True Leaves.

Groups	Palisade Tissue Thickness (μm)	Spongy Tissue Thickness (μm)	Leaf Thickness (μm)	Leaf Midrib Thickness (μm)	Palisade-to-Spongy Ratio	Leaf Structural Density
Xinluzhong 61-CK	47.69 ± 1.75 a	95.22 ± 0.59 a	168.02 ± 4.96 a	162.28 ± 0.62 a	0.50 ± 0.02 b	0.28 ± 0.00 b
Xinluzhong 61-Cold	38.10 ± 0.64 b	54.45 ± 4.22 c	110.10 ± 8.83 c	183.79 ± 32.76 a	0.7 ± 0.06 a	0.35 ± 0.03 a
Tahe 2-CK	41.96 ± 7.5 b	73.82 ± 4.86 b	139.76 ± 9.74 b	128.83 ± 1.59 c	0.57 ± 0.14 a	0.30 ± 0.05 a
Tahe 2-Cold	35.78 ± 3.99 c	58.63 ± 3.25 c	116.19 ± 3.17 c	156.11 ± 7.05 b	0.61 ± 0.03 a	0.31 ± 0.03 a

Note: Data are mean ± SD. Different lowercase letters (a, b, c) within the same column indicate significant differences at *p* < 0.05. a = lowest mean, b = highest mean.

**Table 3:** Effects of Low Temperature Treatment on Stem Anatomical Structure.

Groups	Epidermis Thickness (μm)	Cortex Thickness (μm)	Phloem Thickness (μm)	Xylem Thickness (μm)	Pith Diameter (μm)
Xinluzhong 61-CK	10.69 ± 1.18 a	171.4 ± 47.92 b	142.94 ± 47.77 a	129.14 ± 13.27 b	929.37 ± 58.11 a
Xinluzhong 61-Cold	12.29 ± 1.00 a	164.15 ± 53.11 b	170.74 ± 41.24 b	175.6 ± 10.57 a	1012.67 ± 126.18 a
Tahe 2-CK	11.50 ± 1.41 a	308.62 ± 38.42 a	78.79 ± 1.17 b	193.46 ± 5.68 a	860.94 ± 15.22 a
Tahe 2-Cold	11.12 ± 1.75 a	163.05 ± 31.32 b	77.84 ± 3.93 b	231.71 ± 140.01 a	969.09 ± 155.75 a

Note: The data in this table are presented as mean ± standard deviation. Different lowercase letters indicate statistically significant differences (*p* < 0.05).

## Data Availability

The RNA-seq raw reads of cotton used in this study are currently being submitted to the NCBI Sequence Read Archive (SRA). The accession number will be updated here upon the completion of submission.

## Supplementary Materials

The following supporting information can be downloaded at: https://www.mdpi.com/article/10.3390/ijms27146401/s1.
