# Supplementary material for: Adaptive Structural and Transcriptional Responses Contribute to Cold Tolerance Variation in Xinluzhong 61 and Tahe 2 Cotton Cultivars
Source: Int J Mol Sci. 2026 Jul 18;27(14):6401. doi: 10.3390/ijms27146401 (PMC13410011; doi:10.3390/ijms27146401)

Table S1.Gene expression evaluation by RT-qPCR.

Gene Name	Forward primer	Reverse primer	Acc. Num.
**GhGRF2**	GCCGCTA CTTCACCACG TGA	CGGTTC CATGAA CCTAACC AT	Ghir_A01G001190.1
**GhCYSB**	GCTACTTTGG GTGGAATTAG CC	CGTCTTGTC ACCATCCATC GC	Ghir_D09G011270.1
**GhVPS46.2**	GCTCGATGCCGTTGTTGCTA	ACTCTGCTTGAACCTCCATA	Ghir_A06G020410.1
**GhCYP76C4**	GAAGAACTCGATAGAAATCTG	GTAAACGAGTAACATCAGATT	Ghir_A08G018380.1
**GhNB-ARC**	GTTTGCTGTTCAATTCCTAA	ATTAGTTCCACCCACATAGT	Ghir_D10G024660.1
**GhRST1**	GTTTGCTGTTCAATTCCTAA	ATTAGTTCCACCCACATAGT	Ghir_A09G003090.1
**GhRPL27A**	GTTTTACTGCCCCATCGTCA	CTTCTCAGCAGTCTTAGATA	Ghir_D02G016530.1
**GhGMP**	GGACATTGGGATGAAAATAA	GTTGATTGCCCAATGGAGCA	Ghir_D11G008860.1
**GhCYP82C4**	GGACTCTCAAATTTTCAGTA	CGTTAGCTGCTTACAATGCA	Ghir_A05G026490.1
**GhCYP704A2**	CGGCCAACTCGTCGAAGAAG	GTTGATTGGATTTCAGGGTG	Ghir_A12G025310.1

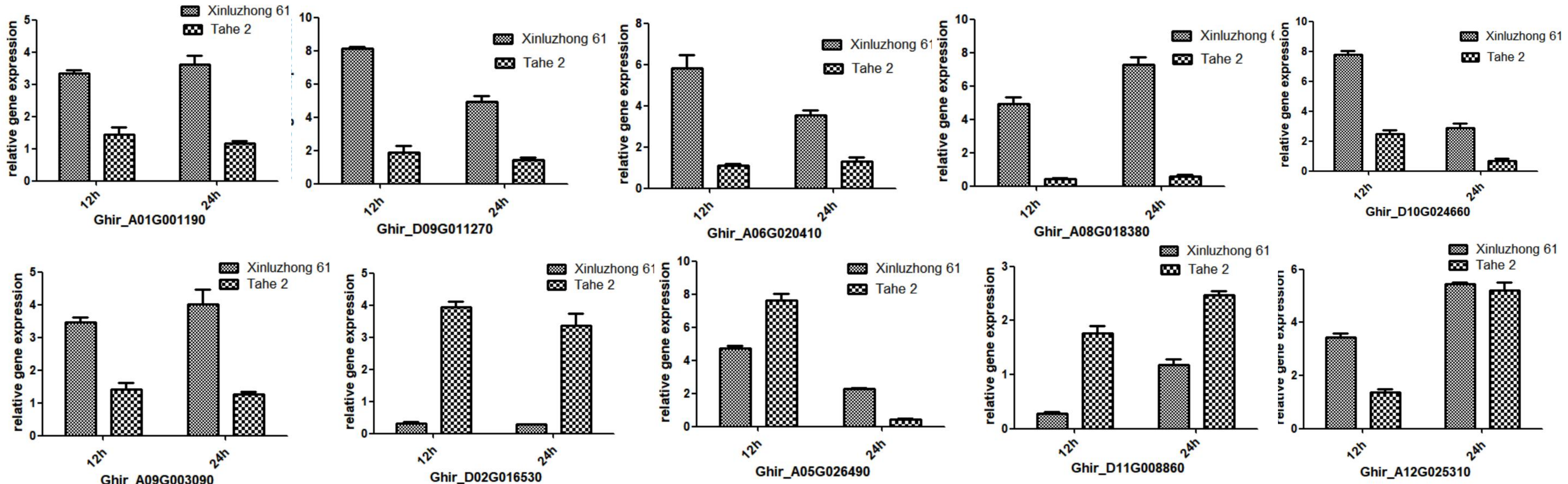

Table S2.Cold-related Gene expression evaluation by RT-qPCR.

Gene Name	Forward primer	Reverse primer
**GhCBF1**	CTGGTAGTGGAAATGGGCGT	CCCAGCCAAATCCTCGACTT
**GhCBF2**	GCGTTAGCCCTGAGAGGAAG	ACCTCTGTTTGCGTCGTTCA
**GhCBF3**	CTCCCAACCATAAACTCCAACT	GACTAGCTCCACTCCCGGAAT
**GmICE1**	GGACGAGGAGATGATGTTGGCTTC	CCCTCACTTCACAAACCCACTTCC
**GhCOR**	AAGTGGGTTTCCGAGGTGAG	GACAAGCCGACCTTCCTCTA
**GhUBQ7**	AGAAGAAGATGCGACGAACG	CCTCGATCGATCGATCGATC

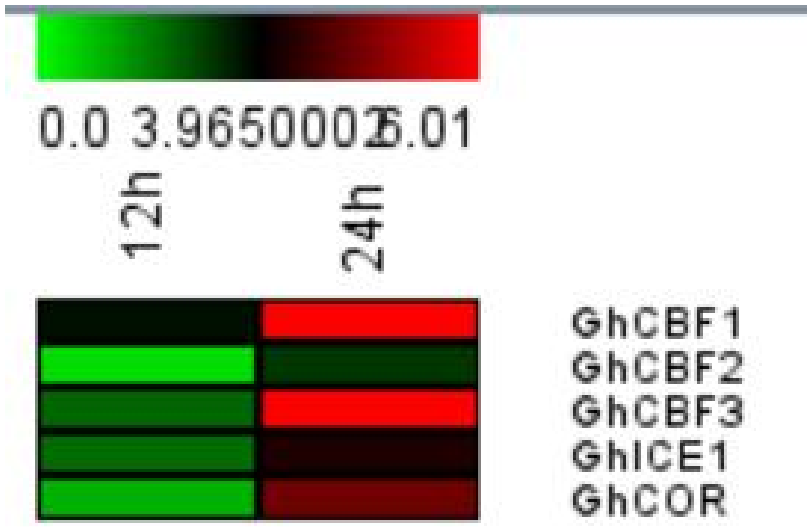

Supplement: Supplementary file 1 [file ijms-27-06401-s001.zip › ijms-4203874-supplementary.pdf]
